# Immature stages of giants: morphology and growth characteristics of *Goliathus* Lamarck, 1801 larvae indicate a predatory way of life (Coleoptera, Scarabaeidae, Cetoniinae)

**DOI:** 10.3897/zookeys.619.8145

**Published:** 2016-09-27

**Authors:** Tomáš Vendl, Petr Šípek

**Affiliations:** 1 Department of Zoology, Faculty of Science, Charles University, Viničná 7, CZ-128 44 Praha 2, Czech Republic

**Keywords:** Afrotropical region, captive breeding, Goliathus, growth trajectories, immature stages, larval development, nutrition shift, rose chafers

## Abstract

The third larval instar of *Goliathus goliatus* (Drury, 1770), *Goliathus orientalis* Moser, 1909 and *Goliathus albosignatus* Boheman, 1857 are described and illustrated for the first time and compared with the immature stages of other Cetoniinae. Larval development of *Goliathus goliatus* is investigated under laboratory conditions, with particular emphasis on food requirements. These results support the obligatory requirement of proteins in the larval diet. The association between larval morphological traits (e. g., the shape of the mandibles and pretarsus, presence of well-developed stemmata) and larval biology is discussed. Based on observations and the data from captive breeds it is concluded that a possible shift from pure saprophagy to an obligatory predaceous way of larval life occurred within the larvae of this genus, which may explain why these beetles achieve such an enormous size.

## Introduction

Goliath beetles (*Goliathus* Lamarck, 1801) are among the largest beetles in the world and undoubtedly the largest of the subfamily Cetoniinae. With their size exceeding 11 cm in the largest males, they have been the focus of entomologists’ interest for centuries. Strangely enough, their systematics, ecological requirements, and developmental characteristics remain largely unknown and have been poorly investigated. Due to their colour polymorphism and suspected ability of hybridization (Wiebes 1968, Lachaume 1983, McMoningle 2006, Meier (undated)) a vast number of taxonomic names was introduced to cover the variability of the several species attributed to this genus (Marais and Holm 1992, Krajčík 1998). The genus is most commonly considered to encompass five species inhabiting tropical forests and savannas of tropical and subtropical Africa (Krajčík 1998, Sakai and Nagai 1998), but Mawdsley (2013) in concordance with Marais and Holm (1992) includes the closely related genera *Argyrophegges* Kraatz, 1895, and *Hegemus* White, 1845 as subgenera of *Goliathus*. Moreover Wiebes (1968) also included members of the genus *Fornasinius* Berlontoni, 1853 in the genus. Neither of these views is supported in this article. Information on the ecology of the genus is somewhat sparse, and available only for *Goliathus albosignathus* Boheman, 1857 (Holm and Marais 1992). Males are generally larger than females, and use their cephalic horns as well as prolonged forelimbs in combat over feeding spots and females. At least some species are known to aggregate at ‘sleeping’ trees at dusk (Holm and Marais 1992). Nothing is known of the immature stages and development under natural conditions, however larvae of the closely related genus *Argyrophegges* are suspected to be associated with the burrows of hyrax species (Mammalia: Procaviidae) (Malec (undated)).

The availability of Goliath beetles to breeders has led to the publication of several breeding manuals, which contain very interesting information on the nutritional requirements of larvae (McMonigle 2006). It is generally believed that the genus is difficult to breed and that a protein-rich diet is necessary for its successful development. Some breeders even report *Goliathus* larvae to be predaceous (McMonigle 2001, 2006, 2012, Meier 2003, Meier and Campbell (undated)). This is in contrast to most other rose chafers, whose larvae are able to develop successfully on a substrate composed only of decaying plant matter; this is also true for some genera with very large beetles (e.g., *Mecynorhina* Hope, 1837, *Mecynorhinella* Marais & Holm, 1992) (Micó et al. 2008, Christiansen 2013). However, no experimental study has been carried out to confirm or disprove the assumptions made in these breeding manuals.

The immature stages of Goliathini have been described in several works (e.g. Sawada 1991, Donaldson 1992, Nogueira et al. 2004, Šípek et al. 2008, Perissinoto and Orozco 2013). Micó et al. (2008) provide a matrix of 38 larval characters for 12 species of this tribe including the larvae of *Goliathus orientalis* Moser, 1909 and *Fornasinius fornasinii* (Berlontoni, 1852), but without a proper description. Larvae of *Hypselogenia geotrupina* (Bilberg, 1817) described by Oberholzer (1959) thus remain the only known and fully described immature stages of the subtribe Goliathina. According to Nogueira et al. (2004), a knowledge of *Goliathus* immature stages is crucial for a better understanding of Cetoniinae phylogeny, and we therefore decided to contribute towards this goal.

The aims of this study are: 1) to describe the third-instar larva of three goliath beetle species – namely *Goliathus goliatus* (Drury, 1770), *Goliathus orientalis* Moser, 1909 and *Goliathus albosignatus* Boheman, 1857 and compare them with larvae of other known Goliathini; 2) to examine larval biology and development, with particular consideration of the importance of proteins in larval growth.

## Materials and methods

### Origin of material and morphological investigations

Larval material was obtained either by direct breeding of wild collected adults by the authors or donated by other scarab breeders for the purpose of this study: 2 last instar larvae of *Goliathus albosignatus* Boheman, 1857 donated by O. Jahn (Czech Republic), having been reared from beetles imported from Tanzania in 2004; 12 last instar larvae of *Goliathus goliatus* (Drury, 1770) reared from adults imported from Cameroon in December 2010; 6 last instar larvae of *Goliathus orientalis* Moser, 1909 donated by O. Jahn (Czech Republic), having been reared from beetles imported from Tanzania in 2004.

The terminology for larval description follows Hayes (1929), Böving (1936) and Ritcher (1966). Antennomeres I–IV were abbreviated in the description with ‘an I’ – ‘an IV’. In order to provide the most accurate information on chaetotaxy, the hair-like setae of the cranium and other structures, were classified by their relative size into two groups: medium to long (80–300 µm) and minute or small (5–40 µm or less). Morphological observations and measurements were made using a Nikon SMZ 745 stereomicroscope and Olympus BX 40 dissecting microscopes, equipped with an Olympus Camedia 5060 digital camera. Photographs were taken using a Canon 70D digital camera, equipped with a Canon MP-E 65/2.8 macro lens with 5:1 optical magnification and a Canon EFs 60/2.8 macro lens for images of larger body parts. Partially focused images of each specimen were combined using Zerene photo stacker software (Zerene systems LLC, Richland, USA). All pictures were digitally enhanced using Adobe Photoshop CS4.

The specimens included in this study are deposited in the following collections:

CUPC Department of Zoology, Charles University, Prague, Czech Republic (Petr Šípek)

NMPC National Museum, Prague, Czech Republic (Martin Fikáček, Jiří Hájek)

### Larval rearing and experimental design

For these experiments, larvae obtained by breeding two pairs of goliath beetles (*Goliathus goliatus*) imported from Cameroon in January 2009 were used. They were kept together in a breeding terrarium (90 × 45 × 55 cm) with a 30 cm deep mixture of soil and leaf litter. The substrate was checked once a week and the newly laid eggs were transferred individually to 500 ml plastic boxes for hatching. The larvae were kept in the same boxes during the entire first and second instar. Third instar larvae were transferred to 1000 ml plastic boxes. Larvae were raised in separate containers during the entire experiment to prevent cannibalism and to allow individual tracking of growth. The breeding substrate was composed of a mixture (1:1) of crushed beech (*Fagus sylvatica*) leaf litter and organic soil (common garden compost). Approximately half of the substrate was replaced with fresh substrate every weighing period. Boxes were kept in a climate chamber at an average temperature of 28°C with a 12:12 L/D cycle. Water was added to the substrate when necessary and the substrate was kept damp but not sopping. The eggs were monitored every other day to determine the date of hatching and newly hatched larvae were randomly divided among three diet regimes.

To examine the dependence of larval development on nutriment, larvae of *Goliathus goliatus* were reared under three different dietary regimes: 1) on substrate with proteins added *ad libitum* (‘fully nourished regime’, 23 larvae); 2) reared on substrate, but proteins were supplied after a period of starvation (‘partly nourished’, 11 larvae); and 3) reared on substrate without the addition of proteins during the entire experiment (‘undernourished regime’, 11 larvae); see below. Some of the larvae were killed at the end of the experiment and used for the study of intestinal microorganisms (Zadrobílková et al. 2016).

The rearing conditions of the initial two instars were identical for all larvae in the experiment. In accordance with the breeding manual (McMoningle 2001, 2012; Meier 2003; Meier and Campbell (undated)) we started to feed all larvae on pellets of soft-moist dog food (FROLIC® Complete with Beef) from the onset of the second instar. These pellets were replaced every weighing session to prevent an excessive growth of mites and other unwanted organisms. In order to monitor the effect of nourishment on larval growth, one cohort of larvae was allowed to continue to feed on pellets (‘fully nourished group’) from the onset of the third instar whereas the other larvae were denied pellets. A part of the unfed larvae were allowed to resume feeding on pellets at a given point after a period of protein deprivation during the third instar (100–240 days after the onset of the third instar). These individuals are referred to as the ‘partly nourished’ larvae. The last cohort of larvae (‘undernourished group’) was raised without a supply of pellets during their entire final instar.

To monitor larval development, we weighed larvae every five days from hatching throughout their entire development using a KERN 450-3M digital scale with a precision to 0.001 g. This weighing interval was chosen in view of the optimal frequency of pellet replacement (McMoningle 2006, 2012; Meier 2003, Meier and Campbell (undated)), which minimizes larval stress and the proliferation of scavenger mites and moulds. For each instar, we determined development time (in days) and maximal mass (in milligrams). In order to compare growth under different feeding regimes in the third instar, we calculated the growth rate at the beginning of the instar and the growth rate just before and after the start of pellet supply to the partly nourished larvae. Growth rate was calculated as a daily mass increment measured over a period of ten days in all aforementioned periods. The initial weight, first recorded at the beginning of the instar was not taken into account because the larvae moult with an empty gut which is refilled after ecdysis, thus growth rate computed with this initial weight would contain false growth caused by new gut content. The recording period before pellet supply was ten days immediately prior to protein addition, after pellet supply it was from the fifth to the fifteenth day after protein addition (pellet supply). The calculating period of growth rate for undernourished larvae was arbitrarily set as the 150^th^ – 160^th^ day after the beginning of the instar which corresponded roughly to the point when the feeding of the partly nourished larvae with proteins began.

### Data analysis

To compare the development times and body mass under the food regimes of the first two instars and the final instar we used a one-way ANOVA and Student’s t-test, respectively. As initial weight is expected to be correlated with growth rate, differences in growth rate were tested using an ANCOVA with the initial weight of the recording period as covariate. Normality of the data was verified using the Kolmogorov-Smirnov test, the Cochran test indicated that variances were homogeneous so no transformations were necessary. The significance level was set to 0.05. Statistical analyses were performed using the program STATISTICA, version 6.0 (StatSoft Inc. 2001).

## Results

### Larval morphology

#### Description of *Goliathus* third instar larvae

Figs 1–3

**Figure 1.**
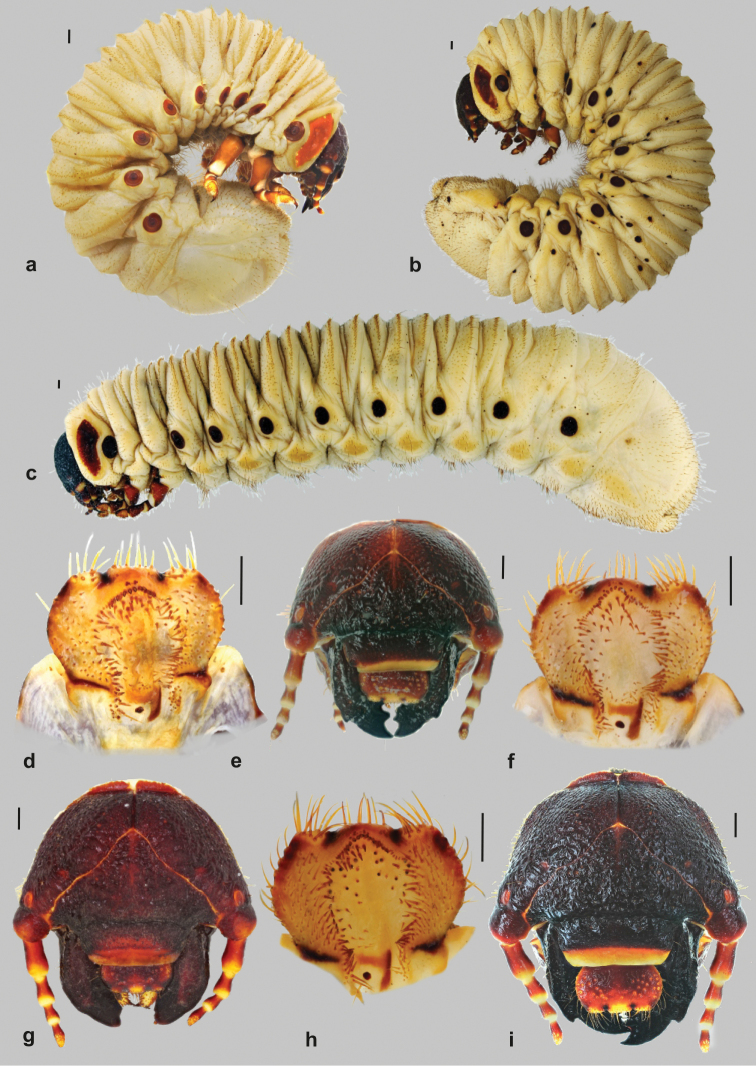
Immature stages of the genus *Goliathus*: **A–C** habitus (**A** *Goliathus albosignatus* **B** *Goliathus goliatus* **C** *Goliathus orientalis*) **D, F, H** epipharynx (**D** *Goliathus albosignatus* **F** *Goliathus goliatus* **H** *Goliathus orientalis*) **E, G, I** cranium (**E** *Goliathus albosignatus* **G** *Goliathus goliatus* **I** *Goliathus orientalis*). Scale bars: 1 mm.

**Figure 2:**
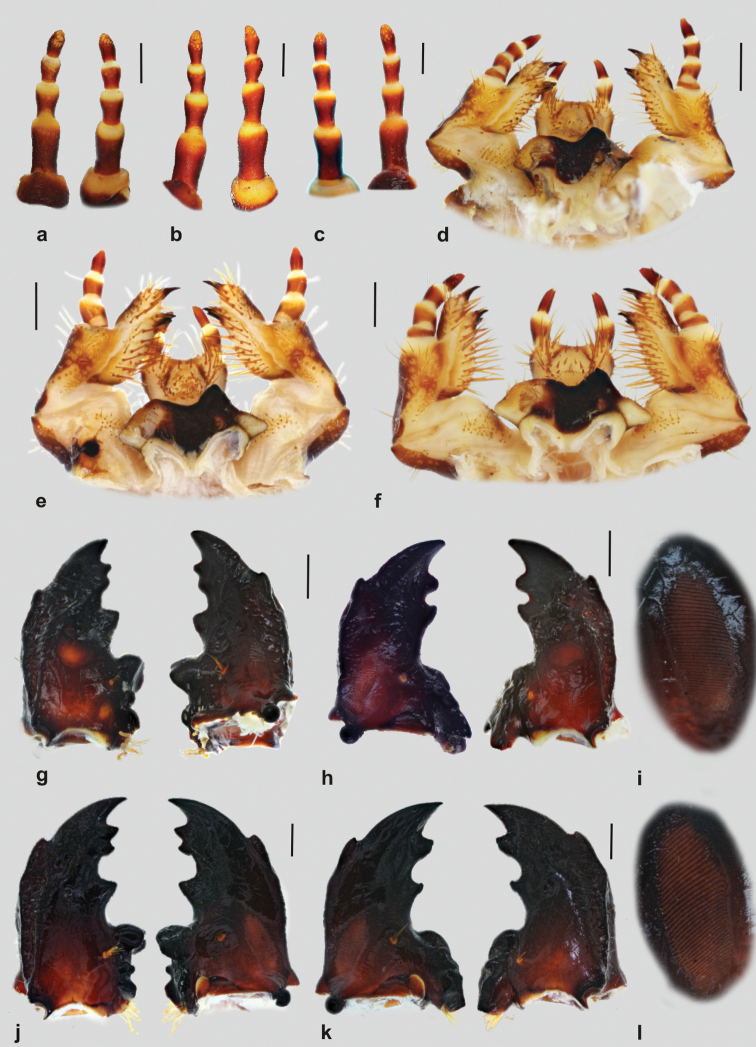
. Immature stages of the genus *Goliathus*: **A–C** right antenna, dorsal and ventral aspect (**A** *Goliathus albosignatus* **B** *Goliathus goliatus* **C** *Goliathus orientalis*) **D–F** maxillo-labial complex, dorsal aspect (**D** *Goliathus albosignatus* **E** *Goliathus goliatus* **F** *Goliathus orientalis*) **G–I** *Goliathus albosignatus*, mandibles (**G** left mandible, dorsal and ventral aspects **H** right mandible, dorsal and ventral aspects **I** stridulatory area **J–L** *Goliathus goliatus*, mandibles (**J** left mandible, dorsal and ventral aspects **K** right mandible, dorsal and ventral aspects **I** stridulatory area. Scale bars: 1 mm.

**Figure 3.**
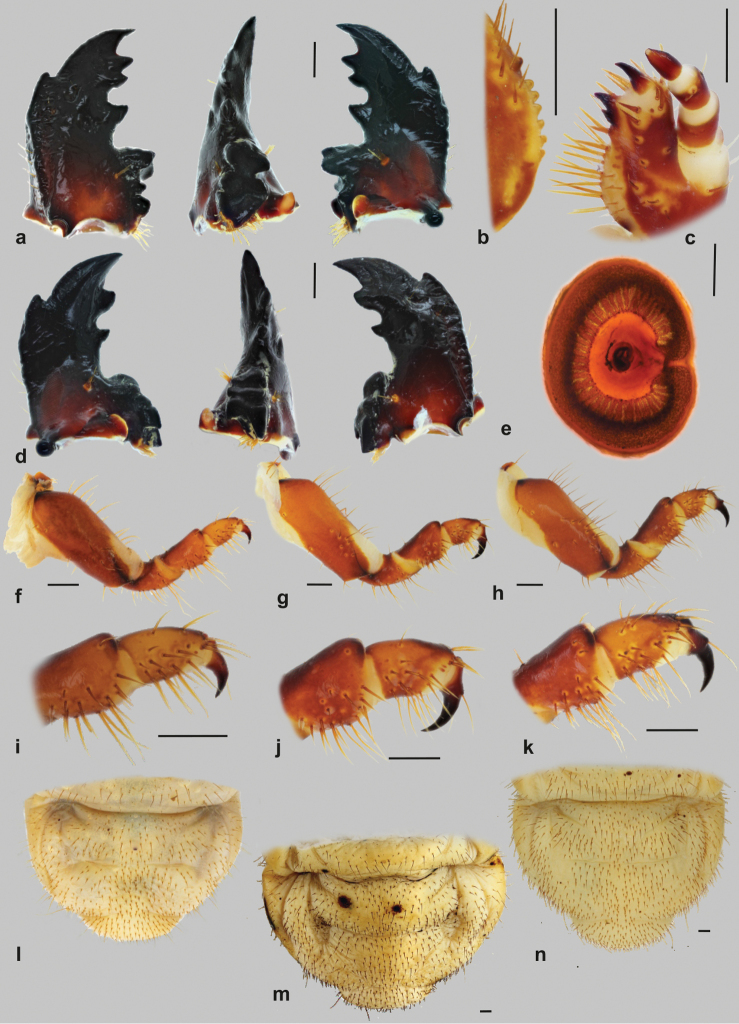
Immature stages of the genus *Goliathus*: **A** *Goliathus orientalis*, left mandible, dorsal, medial and ventral aspect **B** *Goliathus orientalis*, maxillar stridulatory teeth, lateral aspects **C** *Goliathus orientalis*, detail of mala and unci, ventro-lateral aspect **D** *Goliathus orientalis*, right mandible dorsal, medial and ventral aspect **E** *Goliathus albosignatus*, thoracic spiracle **F–H** prothoracic leg (**F** *Goliathus albosignatus* **G** *Goliathus goliatus* **H** *Goliathus orientalis*) **I–J** tibiotarsus and preatarsus (claw) (**I** *Goliathus albosignatus* **J** *Goliathus goliatus* **K** *Goliathus orientalis*) **L–N** raster (**L** *Goliathus albosignatus* **M** *Goliathus goliatus* **N** *Goliathus orientalis*). Scale bars: 1 mm (when not otherwise specified), 0.1 mm (**A, B, C**); 0.5 mm (**D**)

Live larvae straight, unbent, relatively slim, but C-shaped when killed using standard methods. Abdomen 9-segmented; abdominal segments IX and X fused dorsally, ventral border of the respective segments indicated by an incomplete groove. Abdomen relatively slim, segments I–VI proximally of the same size and thickness as thoracic segments II–III, segments VII and VIII usually slightly thickened, last abdominal segment usually much thinner than the preceding one. Length of larvae studied (third instars) 58–150 mm.

**Head capsule** (Fig. 1E, G, I): maximum width between 7.5 and 12.8 mm. Surface of cranium with rugose sculpture, dark brown to black, parts of antennae and anteclypeus yellowish-brown. Frontal sutures bisinuate, more or less warped. Epicranial insertions of antennal muscles distinct. Cranial chaetotaxy summarized in Table 1. Setae of cranium generally indistinct, often broken or worn. Anterior and exterior frontal setae minute, almost invisible (unless observed with particular care). Clypeus subtrapezoidal, membranous anteclypeus taking up nearly 1/3 of entire clypeal area. Postclypeus strongly sclerotized with one anterior and one exterior clypeal seta (often with a minute seta nearby). Frontoclypeal suture distinct. Stemmata present, well developed with optical active layer.

**Table 1.** Cranial chaetotaxy of *Goliathus albosignatus*, *Goliathus goliatus*, and *Goliathus orientalis*.

Group of setae	epicranium				frons				clypeus		labrum				
	DES	PES	AES	EES	PFS	EFS	AFS	AAS	ACS	ECS	PLS	PMS	ELS	LLS	MLL
***Goliathus albosignatus***															
long and medium setae	1	0	1	2–6	1	0	0	1	1	1	4–9	1–2	2	8–9	8–9
minute setae	6–8	6–7	2–7	11–17	5–7	1–4	6–7	(1)	0	0–1	0–4	0 (1)	0	0	0
***Goliathus goliatus***															
long and medium setae	2	1	1	11–13	1	0	0	1	1	1	0	1	3–4	9–12	8
minute setae	3–7	7–10	(1)	8–15	0–3	1–2	1–5	1	0	0	2–4	1–3	0–1	0	0
*Goliathus orientalis*															
long and medium setae	(1)3–4	1–2	1	7–15	1–2	0	0	1	1	1	(1)4–6	1+0-5	3–6	7–8	8
minute setae	3–9	0–3	0–1	7–17	0–3	0–2	0–3	0	0	0–1	0–2	0–2	0	0	0

Abbreviations: AAS = setae on anterior frontal angle ; ACS = anterior clypeal setae ; AES = anterior epicranial setae ; AFS = anterior frontal setae ; DES = dorsoepicranial setae ; ECS = exterior clypeal setae ; EES = exterior epicranial setae ; EFS = exterior frontal setae ; ELS = exterior labral setae ; LLS = setae on lateral labral lobe ; MLL = setae of medial labral lobe , PES = posterior epicranial setae ; PFS = posterior frontal setae ; PLS = posterior labral setae ; PMS = paramedial labral setae . Numbers in brackets indicate a rarely occurring state. For explanation of length categories of setae see ‘Materials and methods’.

**Labrum**: Symmetrical, anterior margin trilobed, with numerous setae and several pores. Clithra present. Dorsal labral surface with several setae organised in irregular rows and groups. Posterior row with approximately 2–6 minute or medium length setae, anterior row with one prominent paramedian and several smaller ones. Lateral margin of labrum with 2–3 prominent setae and another 1–2 medium length setae.

**Antennae** (Fig. 2A–C): tetramerous (an I–IV); relative length of antennomeres: an I > an II > an IV > an III. Ventral and apical projection of the penultimate antennomere III indistinct or entirely absent, the respective sensorium present, but tiny and indistinct. Ultimate antennomere (an IV) beside round apical sensory field with 10–30 dorsal and ventral sensory spots.

**Epipharynx** (Fig. 1D, F, H): Haptomerum: zygum strongly convex (haptomeral process absent), with arched or angulate row of approximately 14–18 stout setae. Another 8–12 stout setae scattered on the inner surface of zygum, typically longer than those arranged in row. Exterior surface of zygum with central group of approximately 8 sensilla (often organised in two paramedian subgroups) and a further two paramedian groups of 3-4 sensilla next to clitra. Proplegmata absent. Acroparia: external margin of medial labral lobe with 8–9 long setae. Lateral labral lobes with 5–6 long setae. Lateral margin of acanthoparia fairly sclerotized, straight or undulated. Acanthoparia with up to ten setae often originating from a tubercle. However, the presence and development of the tubercles as well as the number of setae present on acanthoparia may be variable even on opposite sides of the same epipharynx (possibly due to abrasion). Setae in the proximal third of acanthoparia small, hair-like, the remaining setae equal in size and similar to those of acroparia. Plegmata absent. Chaetoparia asymmetric, exhibiting 6–10 irregular longitudinal rows of setae. Central rows with stout, spine-like setae similar in shape to those of the setae of zygum. Setae in exterior rows of chaetetoparia decreasing in size towards the margin of epipharynx. Right half of chaetoparia with around 70–106 setae, left with approximately 85–115. Dexiotorma straight, relatively narrow, right pternotorma absent. Laeotorma well developed, left pternotorma more or less well developed. Haptolachus: sense cone (left nesium) high, with 4 pores, sclerotized plate absent. Platesclerite large, in shape of reversed Greek letter “Γ”; longitudinal part heavily sclerotized, while transverse part (bordering with pedium) is less sclerotized. Posterior part of haptolachus with only 1-2 pore-like sensilla. Phoba and crepis absent.

**Mandibles** (Figs 2G–L; 3A, D): asymmetrical, narrow. Scissorial part about two times longer than molar part. Scrobis with a row of 4–6 setae; longitudinal furrow deep. Anterolateral portion of dorsal mandibular surface with two prominent setae (which may often be broken). Patches of 4–18 dorsomolar and ventromolar setae concealed in a single rim. Stridulatory area with 29–50 fine transversal ridges, interval between rigdes subequal in entire area. Scissorial area with four and three prominent, sharply pointed teeth on left and right mandible, respectively. The second and third tooth on left mandible fused at base but with well separated apical blade. Exterior margin of both mandibles with prominent, sharply pointed exterior tooth situated approximately at base of its apical third.

Molar lobes of both mandibles with projections. Base of right mandibular calyx bilobed (in medial aspect), dorsal lobe about twice as large as ventral. Calyx of left mandible flattened with arcuate basal margin.

**Maxilla** (Figs 2D–F, 3B–C): dorsal surface of cardo and labacoparia with 8–14 and 20–44 setae, respectively. Stipes dorsal with approximately 35–45 setae, interior stipital setae more or less slender, hair-like; setae stouter and larger towards exterior stipital margin; exterior margin with 2–5 prominent very long and stout setae. Stridulatory area composed of 4–7 feebly sclerotized conical or semi-conical (almost truncate or even abraded in older specimens) stridulatory teeth (Fig. 3B); truncate process low and transverse. Ventral surface of stipes with apical group of approximately 5 hair-like setae. Galea and lacinia entirely fused forming mala, galeo-lacinial suture indistinct, entirely absent on ventral face. Galear portion of mala with single falcate uncus and 15–22 setae in longitudinal rows; setae around apex and on interior row very long and stout. Lacinial part of mala with 2 subequal unci (Fig. 3C), subtriangular and fused at base; larger uncus sometimes with lateral hump, apices of both unci pointing towards each other. Base of unci with 2–3 conspicuous conical setae, one usually very small, not exceeding one third of larger uncus; dorsomedial side of lacinia with ca. 30–40 very long hair-like setae.

Ventral surface of mala markedly sclerotized, apical part with 2 irregular longitudinal rows of 3–4 hair-like setae. Maxillary palps four-jointed, basal joint somewhat reduced on ventral side and retracted into palpifer, thus visible only as narrow sclerotized ring on dorsal face of maxilla, alternatively basal joint entirely retracted into palpifer; penultimate joint of maxillar palpus with 2 setae.

**Hypopharyngeal sclerome** (Fig. 2D–F). Asymmetric, hypopharyngeal process subtriangular, pointed. Row of approximately 30–35 tegumentary expansions (= phoba, *sensu* Böving 1936) present on left lateral lobe. Approximately 10–15 tegumentary expansions present on right central part of scleroma and below its right medio-posterior margin. Lateral lobes feebly to moderately sclerotized, both with approximately 12 hair-like setae.

**Ligula** (Fig. 2D–F). Anterior margin of ligula deeply concave. Dorsal surface with two paramedial oblique sclerotized bar-like areas and paramedial group of approximately 25–35 hair-like setae on each side; posterior and medial setae of this group shorter and stouter. Paramedial pair of prominent setae on anterior margin absent. Labial palpi dimerous.

**Thorax** (Fig. 1A–C). Prothorax with single dorsal lobe, meso- and metathorax with 3 well- developed lobes. Prothoracic sclerite large, well sclerotized, bordered with only few setae at its anterior margin. Chaetotaxy of thoracic sublobes rather sparse. Prothorax: dorsum with only few irregular setae, lacking the typical rows of setae found on dorsal and lateral parts of subsequent segments. Pleural part of meso- and metathoracic sublobes with 3–6 tenuous rows of rather short, hair-like setae, tergal part with 1–3 dense rows of short hair-like to spiny setae, interspersed with a few very long, hair-like setae on some lobes. Thoracic spiracle (Fig. 3E) approximately 2.5 × 1.6 mm, elliptic, heavily sclerotized; respiratory plate C-shaped, arms of lobes approximated, almost concealed. Respiratory plate with numerous tiny holes. All pairs of legs (Fig. 3F–K) subequal. Pretarsi with falcate, sharply pointed claw, bearing 2 basal setae (Fig. 3I–K).

**Abdomen** (Figs 1A–C, 3L–N)): nine-segmented. Segment IX and X fused dorsally, ventral border of the respective segments indicated by a shallow ridge. Dorsa of abdominal segments I–VI with 3 sublobes, segments VII and VIII with 2 sublobes. Chaetotaxy of abdominal segments I–VII similar to those of meso- and metathorax. Abdominal spiracles slightly smaller than mesothoracic one, all spiracles subequal, however spiracles of posterior segments more circular. Dorsum of last abdominal segment sparsely, but evenly covered with short setae, with four tenuous rows of medium long or long hair-like setae.

**Raster** (Fig. 3L–N). Palidium absent (*Goliathus goliatus*) or rudimental, composed of 2 more or less irregular rows of 4–8 shortened obtuse pali. Septula poorly developed or entirely absent. Tegilla fused, composed of numerous evenly distributed short setae, covering almost whole ventral surface of last abdominal segment. Chaetotaxy of ventral and dorsal anal lip similar to those of tegilla, composed of numerous short setae and with approximately 5–10 longer setae.

#### Diagnostic characters of *Goliathus albosignatus* Boheman, 1857

Figs 1A, D, E; 2A, D, G–I; 3 E, F, I, L

The morphology of third stage larva of *Goliathus albosignatus* corresponds to the general morphology of *Goliathus* larvae with the following exceptions: Body length 60–70 mm. Cranial width 7.5–8 mm, cranium brown to dark brown. Antennae with 9–12 and 11–13 ventral sensory spots, respectively. Sensory spots elongate in shape and separated from each other only by a very thin portion of cuticle. The ventro-apical projection of penultimate antennal joint rudimental, the respective sensorium small. Epipharynx with 71–75 setae on right part and 85 on the left part of chaetoparia, respectively. Acanthoparia with 6–8 setae on distinctly swollen tubercles; however, the presence and development of these tubercles as well as the setae of the acanthoparia itself may be variable even in the same epipharynx, probably also due to wear. Mandibles: stridulatory area with 29–37 stridulatory ridges, right mandible with the second and third scissorial tooth nearly equal in size and shape. Brustia of calyx with 10–12 and 17–25 setae on right and left mandible, respectively. Pretarsus (claw) about half as long as tibiotarsus. Raster of abdomen with or without rudimental rows of 4–8 pali.

#### Diagnostic characters of *Goliathus goliatus* (Drury, 1770)

Figs 1B, F, G; 2B, E, J–L; 3 G, J, M

The morphology of third stage larva of *Goliathus goliatus* corresponds to the general morphology of *Goliathus* larvae with the following exceptions: Body length 114–150 mm, cranium width 10.2–14 mm. Antennae with 14–25 dorsal and 21–32 ventral sensory spots, respectively. Sensory spots rounded and separated from each other by a relatively thick portion of cuticle. Ventro-apical projection of penultimate antennal joint absent, the respective sensorium very small. Epipharynx with 90–106 setae on right part and 107–113 setae on left part of chaetoparia, respectively. Acanthoparia variable, with 6–10 setae on tubercles, however these structures may be abraded in older specimens. Mandibles: stridulatory area with 42–45 ridges, right mandible with third scissorial tooth distinctly smaller than second, third tooth of left mandible about the size of second. Calyx of right mandible bilobed, ventral lobe reaching only one third of the size of the dorsal one. Brustia of calyx with 26–30 and 43–50 setae on right and left mandible, respectively. Pretarsus (claw) almost as long as tibiotarsus. Raster of abdomen without rows of pali.

#### Diagnostic characters of *Goliathus orientalis* Moser, 1909

Figs 1C, H, I; 2C, F; 3A–D, H, K, N

The morphology of third stage larva of *Goliathus orientalis* corresponds to the general morphology of *Goliathus* larvae with the following exceptions: Body length of studied larvae: 83–95 mm, but it is likely that the larvae can reach a similar size to *Goliathus goliatus* (i.e., 150 mm). Cranium width 10.8–12 mm. Antennae with 11–17 dorsal and 17–24 ventral sensory spots, respectively. Sensory spots slightly elliptical and not densely aggregated. Ventro-apical projection of penultimate antennal joint absent, the respective sensorium very small. Epipharynx with 87–103 setae on right part and 92–104 setae on left part of chaetoparia, respectively. Acanthoparia variable, with 6–8 setae on tubercles, however these structures may be abraded in older specimens. Mandibles: stridulatory area with 39–49 ridges, left and right mandible with third scissorial tooth distinctly smaller than second. Calyx of right mandible bilobed, ventral lobe reaching approximately one half of the size of the dorsal one. Brustia of calyx with 26–37 and 35–37 setae on right and left mandible, respectively. Pretarsus (claw) almost as long as tibiotarsus. Raster of abdomen with two rows of 2–6 pali.

### Larval growth under different nutritional regimes

Breeding conditions during the first and second instar were identical for all larvae; therefore development times and maximal larval mass of these larval stages are presented as a whole irrespective of the experimental regime (Table 3). Uniformity of the development times and maximal body mass in the first two instars under all three experimental regimes was also confirmed by a one-way ANOVA (p > 0.4 for development times and body mass in both instars). Total development time of the fully nourished larvae was 193 ± 5 days. The larvae spent the longest period of time in the third instar, which lasted roughly twice as long as the previous two instars combined. Also, most of the growth took place in the final instar, as the larvae gained 80% of their final body mass during this time (Table 3).

**Table 2.** Main diagnostic characters for larvae of *Goliathus* species.

Species/character	*Goliathus albosignatus*	*Goliathus goliatus*	*Goliathus orientalis*
cranium width	7.5–8 mm	10.2–14 mm	10.8–12 mm
number of dorsal / ventral sensory spots on antennae	9–12 / 11–13	14–25 / 21–32	11–17 / 17–24
shape of sensory spots on antennae	elongate, separated only by a very thin portion of cuticle	rounded and separated by thick portion of cuticle	slightly elongated, separated by a relatively thick portion of cuticle
left chaetoparia of epipharynx	85	107–113	92–104
third scissorial tooth of right mandible	equal to the second tooth	distinctly smaller than the second tooth	distinctly smaller than the second tooth
third scissorial tooth of left mandible	equal to the second tooth	equal to the second tooth	distinctly smaller than the second tooth
calyx of right mandible	ventral lobe about half of the size of the dorsal lobe	ventral lobe about one third of the size of the dorsal lobe	ventral lobe about half of the size of the dorsal lobe
left brustia of calyx	14–23	45–50	35–37
relative length of tarsungulus (claw)	about one half of the length of tibiotarsus	subequal to tibiotarsus	subequal to tibiotarsus

**Table 3.** Summary of the instar-specific developmental characteristics. The values are given as mean ± SE.

Instar	Feeding regime	Development time (days)	Maximal weight (mg)	N
1		35.5 ± 0.88	655 ± 19	45
2		55.1 ± 1.8	5825 ± 132	45
3	fully nourished	104.4 ± 3.36	28712 ± 860	23
	partly nourished	n/a	20412 ± 1273	11
	undernourished	> 197 ± 17	9638 ± 551	11

Food manipulation had a considerable effect on survival and growth. None of the eleven starved larvae pupated, whilst 20 out of the 23 larvae (87%) reared under the fully nourished regime and four out of the eleven larvae (36%) reared under the partly nourished regime pupated; this difference was statistically significant (two-tailed Fisher’s exact test: p < 0.01). On the other hand, all larvae died during the prepupal stage in the pupal cell.

**Figure 4.**
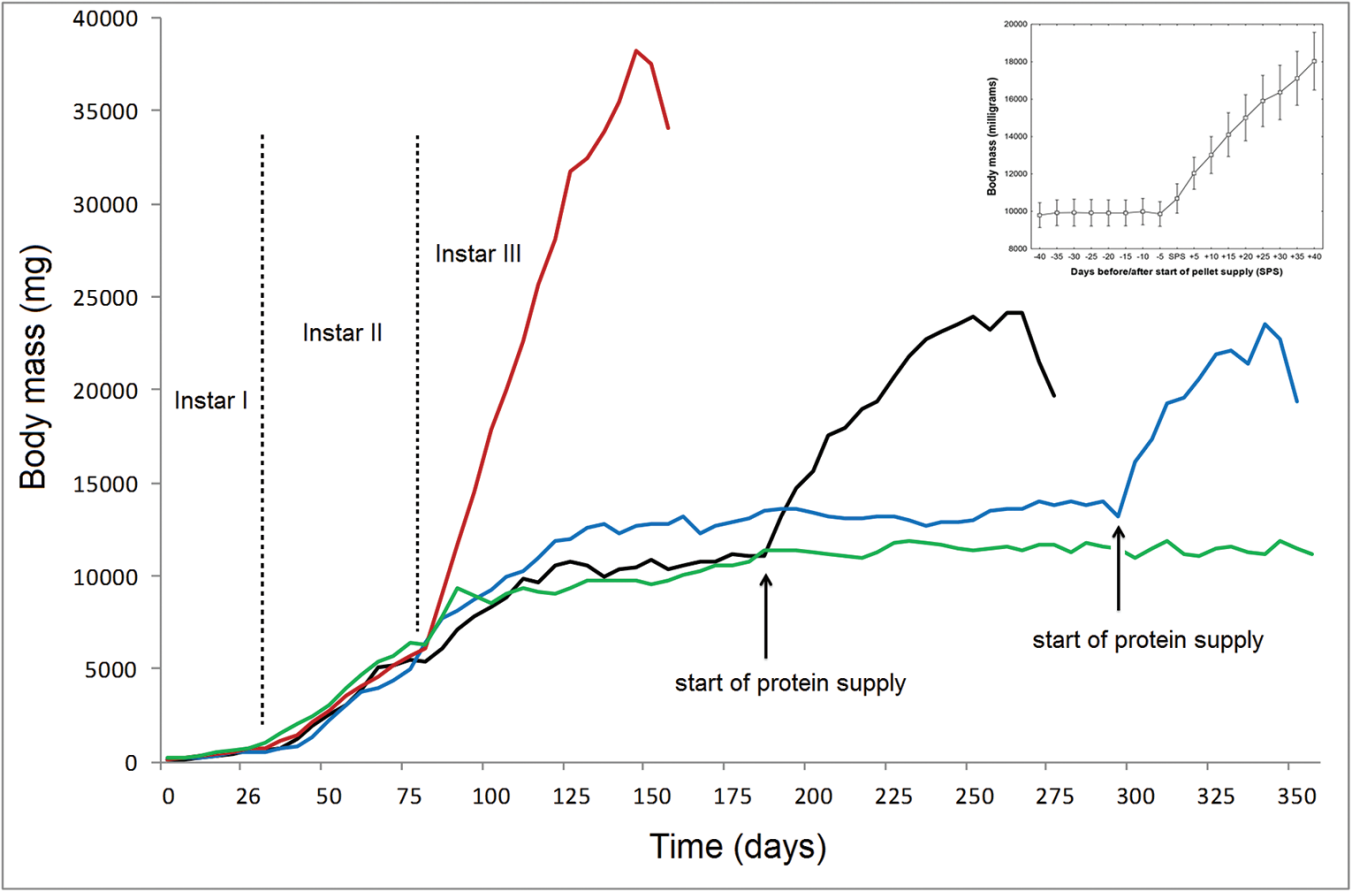
Individual growth trajectories of the fully nourished larva (red line), partly nourished larvae (black and blue lines) and undernourished larva (green line). Evidently, the absence of proteins in larval diet had profound consequences on development. In the third instar, the starved larvae were able to resume growth immediately after the addition of protein to their diet. The inset image shows mean growth of all eleven partly nourished larvae 40 days before and after pellet supply (SPS), irrespective of actual time of pellet supply. Means ± standard errors are depicted.

In the third instar, there were clear differences in growth trajectories between the breeding regimes (Fig. 4). Whilst fully nourished larvae grew regularly till construction of the pupal cell, larvae reared under the other two regimes grew only for a short period after the onset of the third instar (although considerably slower than fully nourished larvae; F_1, 42_ = 99.1, p < 0.001; see also Table 4). The larvae subsequently stopped growing and remained around the reached weight (9969 ± 435 mg; the values were not significantly different between the partly nourished and undernourished regimes: t_20_ = 1.07, p = 0.42). Nevertheless, after the addition of pellets to their diet, the partly nourished larvae were able to resume growth, although their final body mass (t_32_ = 5.45, p < 0.001; see also Table 3) and growth rate (F_1,31_ = 20.9, p < 0.001; see also Table 4) were significantly lower than those of the fully nourished larvae.

**Table 4.** Growth rates (in mg/day) of the differentially fed larvae at the start of the final instar/ before and after protein addition to the starved larvae. The values are given as mean ± SE.

Feeding regime	Start of instar	Before protein supply	After protein supply	N
fully nourished	372.3 ± 26.8	n/a	n/a	23
partly nourished	69.4 ± 8.6	- 0.35 ± 7.7	232.5 ± 27.3	11
undernourished	58.5 ± 7.2	- 0.69 ± 4.9	n/a	11

## Discussion

### Morphology

In the character matrix of 38 larval features published by Micó et al. (2008) no distinct character distinguishing *Goliathus* larvae from the other 41 cetoniinae taxa was identified (except for the evidently wrongly coded character 34/0 – abdominal segments IX and X not fused). Among the other characters shared with only a few other Goliathini or Cetoniinae larvae were the number of respiratory holes on the thoracic spiracle (state 30/2 – more than 60; shared with *Mecynorhina polyphemus* (Fabricius, 1781) and *Fornasinius fornasinii*) and the pretarsi with a sharply pointed claw bearing 2 basal setae (character state 32/0, shared with members of Valgini, Trichiini, Schizorhinini, Cremastocheilini, *Fornasinius fornasinii* and *Dicronocephalus wallichi* Hope, 1831). Similar looking pretarsi have also been described for *Hypselogenia geotrupina* (Oberholzer 1959), *Agestrata orichalca* (Linnaeus, 1769) (Zhang 1984) and members of Taenioderini (Vendl et al. 2014). While the number of respiratory holes may be a size-dependent character, the shape of the pretarsus deserves a more detailed investigation. A falcate claw with two setae at the base clearly represents an ancestral/plesiomorphic stage of pretarsal morphology in the entire plant feeding lineage of scarab beetles (Ritcher 1966, Sawada 1991) but its morphology varies considerably. There are species/groups with a highly developed claw (e.g., the apical part distal to the setae is longer than the proximal part, e.g., the Trichiini), or there are species with an extremely reduced apical portion of the claw (most Taenioderini) and all possible transitional stages occur. McMonigle (2006) argued that the claws of the genus *Goliathus* are retractable and compared them with the claws of Mecynorhina (Mecynorhina) torquata (Drury, 1782) and Mecynorhina (Megalorrhina) harrisii Westwood, 1847. He stated that only the (sub)genus *Megalorrhina* possess claws similar to *Goliathus*, although less developed. We were not able to confirm that the claws of *Goliathus* are “retractable”, but it is clear that the claws are capable to fold back against the tibiotarsus, which seems unusual among pleurostict scarabs. In general we may state that the development of the pretarsal claw in *Goliathus* is exceptional, in *Goliathus goliatus* and *Goliathus orientalis* the claw is even equal in length to the tibiotarsus.

Other distinct characters of *Goliathus* larvae include the extraordinarily coarse surface of the cranium and the extremely well developed stemmata (larval eyes). The most striking feature is the general habitus of living larvae which are straight (Fig. 1C), unbent and have a relatively slim appearance (e.g., the posterior part of the abdomen is more or less as thick as the other parts of the abdomen and thorax) compared with the size of the imago. The characteristic C-shaped form, which is common to most other scarab larvae (living, non-moving), is not present here (unless killed and preserved using standard methods (Figs 1A, B)). Several other larvae of Coryphocerina (Cetoniinae: Goliathini) also have a similar slim appearance, but tend to be C-shaped when in a resting position in substrate (personal observation). The extremely well-developed mandibular scissorial teeth of *Goliathus* larvae (the first tooth is falcate) are also rather extraordinary and exceptional among Cetoniinae (although with this character more caution needs to be exercised as these teeth are often abraded in older larvae). Similarly, the sharply pointed external tooth of the mandible may be another unique character, although there are species with a similar but more or less blunt tubercle: *Hypselogenia geotrupina*, *Ichneostoma pringlei* Perissinotto et al., 1999, *Rhomborhina polita* Waterhouse, 1875 (Oberholzer 1959, Sawada 1991, Perissinotto et al. 1999) and others.

### Species-specific characters

Several species-specific characters have been identified in the immature stages of *Goliathus albosignatus*, *Goliathus goliatus*, and *Goliathus orientalis*, most of them distinguishing *Goliathus albosignatus* from the other two species (see Table 2), which may be due to the different size range of this species. Nevertheless a few characters distinguishing *Goliathus goliatus* from *Goliathus orientalis* have been identified which is quite surprising as these species are closely related and even hybridizing to the F1 generation in captivity (McMonigle 2006, Meier and Campbell (undated)). The validity of these characters still needs to be confirmed, but they might be regarded as additional support for the current separation of the species-level classification of *Goliathus goliatus* and *Goliathus orientalis*.

### Development and nutrition

Although there are no data available on larval biology and development of goliath beetles in the wild, thanks to the long-standing efforts of beetle breeders some interesting findings about their developmental requirements in captivity are available. One of these is the presumed obligatory requirement of proteins in larval diet during its development (McMonigle 2001, 2006; Meier 2003; Meier and Campbell (undated)). Our results confirm this statement. The growth rate of larvae without added protein clearly slowed down immediately after the exclusion of protein pellets at the beginning of the third instar and from a certain point they were not able to achieve a higher body weight (the average weight after ecdysis was around 6 g, while the threshold weight when the larvae ceased their growth was around 10 g). This was in contrast to larvae which were fully nourished with proteins, most of which were able to construct a pupal cell. After the addition of protein pellets to their diet the larvae clearly responded by resuming growth and, interestingly, some of them even constructed a pupal cell, some of them after up to 200 days of starvation. However, all larvae in our experiment died in the pupal cell, possibly due to a high rearing temperature. It is not clear how long the larvae could live without a protein diet, but some of them were still alive 250 days after the beginning of the third instar, when the experiment was terminated and the larvae were inspected for intestinal microorganisms (see Materials and methods). Some of the breeding manuals state that from a certain point in time the larva does not consume the substrate and feeds purely on protein pellets (Wong 2008). Nevertheless, in our experiment the starved larvae produced an amount of faecal pellets comparable to the fully nourished larvae. It is possible that in adverse conditions, when the larva cannot find a suitable source of nutrients, it feeds solely on substrate and waits for more favourable conditions when prey becomes available.

### Conclusions

It has been suggested that goliath beetle larvae are carnivorous and prey on the larvae of other rose chafers in the wild (McMonigle 2001, 2006, 2012; Meier 2003). Indeed, in captivity goliath beetle larvae readily consume the larvae of other common species (e.g., *Pachnoda*; Klátil and Vrána 2008, personal observation). It is also known that other rose chafer larvae (e.g., *Eudicella*, *Cheirolasia*, etc.) enhance their diet by feeding on rose chafer larvae of other species or are even cannibalistic (Klátil and Vrána 2008, Micó et al. 2008, personal observation), but this behaviour is only facultative and the larvae are able to finish their development normally without protein input (Christiansen 2013, personal observation). The possible dependence on live prey may also be reflected in their larval morphology. Mandibles with sharp scissorial teeth (Figs 2, 3), an additional pointed tooth on the lateral face of mandible, legs with conspicuously long and pointed claws (Fig. 3J–K), and well developed stemmata may possibly be linked to a predatory way of life. It is also possible that thanks to a protein rich diet goliath beetles develop considerably faster than comparably sized scarab beetles such as *Megasoma* or *Dynastes*, which thrive well on a “classical” substrate. The development of these species normally takes up to two or three years (Glaser 1976, Morón and Deloya 2001, Klátil and Vrána 2008). However, it can be considerably faster if fed with protein-rich pellets (McMonigle pers. comm.). In this experiment, the regularly fed larvae developed in less than 200 days on average, although their maximal weight was considerably lower than the commonly reported weight (up to 100 g in large males and 50 g in females). The alleged association of the closely related *Argyrophegges* larvae and hyrax (Mammalia: Procaviidae) may indicate that other representatives of the subtribe Goliathina also have alternative larval feeding strategies which may be helpful in achieving their exceptionally large size.
